# “Wearables on vogue”: a scoping review on wearables on physical activity and sedentary behavior during COVID-19 pandemic

**DOI:** 10.1007/s11332-021-00885-x

**Published:** 2022-01-07

**Authors:** Rohit Muralidhar Panicker, Baskaran Chandrasekaran

**Affiliations:** Department of Exercise and Sports Sciences, Manipal College of Health Professions, Manipal Academy of Higher Education, Manipal, Karnataka 576104 India

**Keywords:** Physical activity, Wearable, Smartwatch, Sedentary behavior, COVID-19, Lockdown

## Abstract

**Background:**

Wearables are intriguing way to promote physical activity and reduce sedentary behavior in populations with and without chronic diseases. However, the contemporary evidence demonstrating the effectiveness of wearables on physical health during the COVID-19 pandemic has yet to be explored.

**Aim:**

The present review aims to provide the readers with a broader knowledge of the impact of wearables on physical health during the pandemic.

**Methods:**

Five electronic databases (Web of Science, Scopus, Ovid Medline, Cumulative Index to Nursing and Allied Health Literature and Embase) were searched. The eligibility criteria of the studies to be included were based on PICOT criteria: population (adults, children and elderly), intervention (wearable, smartphones), comparison (any behavioral intervention), outcome (physical activity or sedentary behavior levels) and time frame (between December 1st, 2019 and November 19th, 2021). The present scoping review was framed as per the guidelines of the Arksey and O’Malley framework.

**Results:**

Of 469 citations initially screened, 17 articles were deemed eligible for inclusion and potential scoping was done. Smartphone-based applications with inbuilt accelerometers were commonly used, while a few studies employed smart bands, smartwatches for physical health monitoring. Most of the studies observed the increased use of wearables in healthy adults followed by elderly, children and pregnant women. Considerable reduction (almost—50%) in physical activity during the pandemic: daily step count (− 2812 steps/min), standing (− 32.7%) and walking (− 52.2%) time was found.

**Conclusion:**

Wearables appears to be impending means of improving physical activity and reducing sedentary behavior remotely during the COVID-19 pandemic.

**Supplementary Information:**

The online version contains supplementary material available at 10.1007/s11332-021-00885-x.

## Introduction

COVID-19 has emerged as a public health emergency affecting millions of people’s physical, psychological, and social well-being [1]. State and national governments have enforced lockdowns, home confinements, restrictions on social gatherings and a ban on sports or exercise amenities to contain the spread of the virus [2]. However, the above protective measures have adversely affected the physical and mental health of the global population. Further remote work and virtual classrooms have intensified the physical inactivity and sedentary behavior in the community [3]. Physical inactivity is the inability to meet the global recommendations of 150–300 min of moderate-to-vigorous exercise per week, while sedentary behavior is defined as any waking activity that is characterized by a low energy expenditure (i.e., less than 1.5 METS) [4]. Individuals with high physical inactivity and sedentary behavior are found to have a greater risk of chronic cardiometabolic diseases such as ischemic heart diseases, cancer, obesity, and early mortality [5, 6]. Growing evidence states that physical inactivity and sedentary behavior have substantially increased during the pandemic due to work from home and high screen time [7, 8]. Contemporary evidence suggests that high sedentary time is associated with increased cardiometabolic disease risk independent of weekly physical activity time. Hence, lifestyle interventions focusing on reducing physical inactivity and sedentary behavior are perceived as intriguing measures to prevent the chronic disease risk.

### Wearables in promoting physical activity and reducing sedentary behavior

A wearable is defined as “any body-worn computer that provides useful services while the user performs other tasks”, and includes pedometers, smartwatches, smart wears and activity trackers [9]. The growing popularity of the Internet of Things and technologically sound users have raised the market value of wearables by USD 32.63 billion, and compound annual growth is projected as 15% from 2020 to 2027 [10]. By real-time visualizing, measuring and addressing sedentary behavior and physical activity bouts, wearables continue to increase in popularity and accessibility to the extensive data at the population level [11]. Difficulties in the raw data collection and transformation based on ‘phantom’ algorithms make commercial fitness trackers less reliable and valid than research-based accelerometers [12]. Despite its moderate accuracy, consumer-based wearables continue to be widely used to encourage physical activity and reduce sedentary behavior through behavior change techniques such as goal setting, prompts, cues, self-efficacy and social support [13].

Though wearables use became inevitable in contemporary men and women, the advocacy of wearables for promoting PA and reducing sedentary behavior during these unprecedented times is relatively unknown. Our aim was to provide a comprehensive overview of the breadth and results of studies examining the impact and extent of wearable use on the measurement, encouragement and monitoring of physical activity and sedentary behavior in individuals during successive lockdowns. We aimed to describe the wearables used in the promotion of physical activity (sensors to smartphone technology) as an initial contribution to the informative implementation in practice. We conducted a scoping review with the following objectives:

- To investigate the extent to which the wearables are being used to promote physical activity in the community
- To collate the evidence regarding the prevalence of physical inactivity and sedentary time in individuals measured and monitored using wearables.

## Methods

The present scoping review followed the guidelines of Arksey and O'Malley framework, which consists of five mandatory stages: (1) identifying the research questions; (2) identifying relevant studies; (3) study selection; (4) charting the data; (5) collating, summarizing, and reporting the results [14, 15]. Scoping reviews share similar characteristics of systematic review except for the provision of a broader overview rather than answering a focused question.

### Identifying the research question

Our present scoping review was initiated with a question “What is the breadth of wearable use, its physical and social impact on people during an ongoing pandemic?” The present scoping review aimed to collate and summarize the contemporary evidence investigating the use of the wearables in promoting physical activity and the barriers during the COVID pandemic.

### Identifying the relevant studies

We searched five electronic databases (Web of Science, Scopus, Cumulative Index to Nursing and Allied Health Literature, Ovid Medline and Embase) for the studies investigating wearables and their use to reduce sedentary behavior and improve PA in healthy and diseased populations during COVID-19 pandemic. We searched with the possible combinations of the MeSH terms “wearable activity trackers”, “wearable activity monitors”, “activity monitors”, “activity trackers”, “fitness trackers”, “wearable fitness devices”, “wearable technology”, “wearable devices”, “Fitbit”, “physical activity”, “physical exercise” “aerobic exercise”, “sedentary behaviour”, “sedentary time”, “sedentary lifestyle”, “physical inactivity” with several combinations of Boolean operators and wildcards. We limited our search to a time frame of December 1st, 2019, to May 10th, 2021. The search was again updated on November 19th, 2021. The sample search strategy is provided as an additional file.

### Study selection

We included studies that have: (1) advocated any wearable or digital device; (2) investigated physical activity or sedentary behavior; (3) included only adults; (4) both healthy and diseased; (5) conducted within the timeframe (from December 1st, 2019, till May 10th, 2021. However, the search was again updated on November 19th, 2021); (6) both experimental and observational studies and (7) published only in English. The study selection was based on the PICOT framework, as presented in Table 1.

**Table 1:** Eligibility criteria by which potential studies included based on the PICOT criteria

Variable	Eligibility criteria for the studies to be included
Population (P)	Adults with or without chronic diseases in whom the wearables were employed to assess change in sedentary behaviour or physical activity
Intervention (I)	Studies should have specifically advocated or observed the effects of wearable devices (Fitbit, Polar global positioning system, smart bands such as honor, Huawei, and smart wear)
Comparator (C)	Control group with or without standardized monitors such as pedometers and accelerometers
Outcomes (O)	Step count, step count, sitting time, moderate-to-vigorous physical activity—measured through subjective or objective means
Time frame (T)	From December 1st, 2019, till May 10th 2021. Updated again at November 19th 2021

### Charting the data

We extracted the following variables to a bespoke extraction sheet: author, year, country, design, the objective of the study, type of the wearable, physical activity and sedentary behavior (step count, step time, sitting time, and moderate-to-vigorous activity time, standing time, calorie expenditure). The measurement errors and the authors attempt for appropriate correction were extracted. Furthermore, the sociodemographic influence on the measurement, if any, was recorded.

### Collating, summarizing, and reporting the results

The evidence of the wearables and the associated physical, social and physiological effects were analyzed as qualitative analysis and synthesized narratively to provide the readers and policymakers with the broader knowledge of wearables and their physical, social and mental impact during the pandemic. The data extracted from the search results are provided in Table 2.

**Table 2:** Characteristics of the included studies that investigated the wearables impact on physical health during the COVID-19 pandemic

References	Country	Objectives of the study	Study design	Participants	Eligibility criteria	Time frame	Wearables	Physical health measures	Key findings
Ammar [16, 17]	Germany	To assess the effect of the lockdown on the social and physical health To assess the technology use for diet and physical health	Multi-centric and multi- national survey (ECLB- COVID-19 study)	1047 participants from North Africa, western Asia, Europe and other continents	Adults more than 18 years old without any underlying cognitive impairment	March–April 2020	Global positioning systems, real-time monitoring of mobile devices (fitbit, apple watches, smart bands), mobile phone applications, digital recorders/cameras, and wearables	Daily movement patterns, physical activity in the form of step count and calorie expenditure Apart from wearables, International Physical Activity—Short Form was also administered along with other social and psychological questionnaires	Social and physical activity participation reduced by 42% and 24% Technology use behavior increased by 8.8% before and after lockdown Higher scores for technology-based physical activity promotion was registered than the communication and dietary purposed
Ang IYH [18]	Singapore	To evaluate the effectiveness and feasibility of a personalized m-health program in improving glycaemic control	Single group pre-post trial	Participants with diabetes from Singapore Armed Forces	Full time service professional Type 2 diabetes and pre-diabetes	February–June 2020	Customized mobile application Participants logged their physical activity and dietary intake Health coaching led by dietician and fitness coach for three months	Self-reported measure of duration and frequency However the physical activity prescription by a fitness coach remains unclear	21 Participants completed the study mean HbA_1c_ decreased from 7.6 to 7.0% Mean weight decreased from 75.0 to 73.0 kg
Buoite Stella [19]	Triesta, Italy	to investigate behavioral changes assessed through smart technology devices and the health effects during the COVID-19 lockdown	Online survey	403 Italian residents with and without chronic conditions not limiting the physical activity	Age > 18 years Italian residency Both heathy and with morbidities not limiting physical activity Should be associated with the workplace for the next 12 months	Twice (time frame not mentioned) with 10 days apart	Smart technology device use: smartphone, smart band, smart watch Wear time Mean daily step count for 7 days, mean daily heart rate and peak heart rate	Domains household, occupational Structured physical activity: Gym, pool or sport club Dimensions: frequency and duration of physical activity International Physical Activity Questionnaire—cut off 700 METS or 10,000 steps	197 participants had valid smart technology mean daily step count decreased from 8284 ± 4390 steps to 3294 ± 3994 steps during the lockdown mean HR_peak_ decreased from 61.3 ± 18.2% to 55.9 ± 17.3% METs estimation was 3101 ± 3815 MET, dropped to 1,839 ± 2,254 Wearables can track physiological parameters well
Capodilupo [20]	United states of America	To investigate the impact of physical distancing restrictions on the exercise dimensions and the physiological parameters such as heart rate variability and resting heart rate	Retrospective Analysis	5,436 participants from WHOOP wearable device database	Should have recorded sleeps for at least 120 of the 135 (89%) days between January 1 and March 9 in 2019 and 2020, respectively; and (2) be between the ages of 18 and 80 on May 15th, 2020, when data was extracted for analysis	Baseline: January 1, 2020—March 9, 2020 Post social distancing: March 10, 2020—May 15, 2020	Wearable device (WHOOP strap) measured sleep and physical activity The data extracted from the mobile device application and analyzed using cloud platform	Sleep, resting heart rate and heart rate variability were measured Exercise domains and dimensions, sleep onset, offset, resting heart rate and heart rate variability were measured	Sleep is 15 min later than baseline during the lockdown period Exercise frequency decreased in younger adults whereas decreased in middle aged and elderly population Population spent lesser time in moderate and high intensity activities HRV increased during physical distancing
Ding et al. [21]	China	To measure the change in physical activity during and after lockdown To explore the determinants associated with daily step count during and after the lockdown	Prospective cohort study	815 participants (> 18 years) from 11 workplaces in Pudong District, Shanghai and followed for 202 days	Age > 18 years Should be associated with the workplace for the next 12 months	Twenty-eight weeks	WeRun, a social fitness plugin in WeChat WeRun imports step count data from smartphone inbuilt accelerometers Highly valid (*r* = 0.766) with Actigraph (hip worn accelerometers)	WeRun, a special plugin for a social media-based application “WeChat”, measured daily step count The step count was transferred to the cloud server from a smartphone-inbuilt-accelerometer	Step count reduced in lockdown (3796 steps/day) compared to pre-lockdown (8000 steps/day) Per-day step count gradually increased (+ 34 steps/day) each day during the lockdown Step count attenuated sharply during the lockdown in age: 40 years above
Hamasaki et al. [22]	Japan	To summarize the current evidence regarding the impact of the COVID-19 pandemic on physical activity and sleep measured by using wearable activity trackers	Narrative review	Nine studies that looked at the physical activity among 750,783 people with and without disorders	Not applicable	Search was up to August 2021	Variable wearable devices: Polar, Withings, WHOOP strap, Fitbit, Garmin, PAMSys pendant	Daily step count, walking, standing percentages, Physiological parameters—heart rate and sleep duration	Vigorous intensity exercise did not change however moderate intensity reduced between the lockdown median physical activity per day was significantly decreased from 134.7 min/day during pre-lockdown to 113.9 min/day during post-lockdown There is a need for standardization of wearable devices for measurement of physical activity
Henriksen et al. [23]	Norway	To develop a wearable device or consumer tracker system for surveillance of physical activity during pandemic	Experimental and a cross-sectional study	35 volunteers during the development phase and 130 during the intervention phase	Owned an activity tracker from Garmin, Fitbit, Withings, or Oura willing to share physical activity data	October 2020	Surveillance system extract and assess the data from the consumer tracker (mSpider mobile application)	Steps, energy expenditure Moderate-vigorous physical activity sleep	113 volunteers completed online survey Participants walked 797 fewer steps per day in March, 2020, compared to March 2019 Mean activity energy expenditure was 74 kcal/day lower in March, 2020, compared to March 2019
Jiwani et al. [24]	USA	To assess the acceptability and user inferences on wearable technology intervention in overweight/ obese elderly with type 2 diabetes mellitus patients	Qualitative analysis of a pilot study	Twenty community-dwelling overweight/obese older adults (65 and older) with T2D	Aged 65 years and above self-reported T2D diagnosis overweight/ obese (BMI 25), owning a smartphone	Six months	Fitbit, Smartphone-based applications for self-monitoring	Program Acceptability Logistics Adherence to the diabetes management Impact of wearable on the intervention Perceptions about wearable Impact of the program Challenges faced	High acceptability and adherence with the Fitbit were observed Wearables increased knowledge of health behaviors (tracking physical activity, goal setting and motivation) Personal fitness devices can be used for improving self-efficacy
Kouis et al. [25]	Greece	To quantify physical health changes during COVID-19 lockdown in schoolchildren with asthma using wearable sensors	Observational study	108 asthmatic children, (53 in Cyprus and 55 in Greece)	Participants were eligible if they had a physician's diagnosis of asthma	Not applicable	Wearable watches, global positioning sensors, pedometer	Daily step count reduced at each of the three levels of lockdown level measured from wearables Time spent at home	Mobility reduced from 8996 steps/day to 6499 steps/day after the lockdown Continuous and objective real-time data can be acquired may inform stakeholders about compliance with public health interventions
Mishra et al. [26]	USA	To examine changes in mobility performance in community-dwelling elderly To explore the association between changes in mobility performance and depression during Covid-19 lockdown	Longitudinal study	Ten community older adults were recruited from an ongoing study that investigated fall risk using a wearable pendant sensor	Community-dwelling elderly Age > 75 years Age > 65 years older with a high risk of falling Self-reported fall risks within the past 12 months	Six months (baseline, third and sixth month)	Pendant wearable sensor (PAMSys™, BioSensics LLC, Watertown, MA, USA), worn around the neck	Daily step count reduced Cumulated posture: Sitting and standing Sleep quantity Postural transitions The intensity of physical activity	Decreased standing (32.7%), walking (52.2%) and postural transitions (44.6%) 55% increase in sedentary time 150% increase in depression Increased depression score was correlated with the prolonged sitting bout, nighttime sleep duration, and cadence Reduced sleep time is associated with a 52% increase in depression 18% decreased daily step count in elderly
Niela-Vile´n et al. [27]	Finland	To examine daily patterns of well-being (physical activity, stress, sleep) in pregnant women before and during the COVID-19 pandemic	Longitudinal study	38 singleton pregnant women	Singleton pregnancy Gestational weeks 12–15 Should have a smartphone with Android or iOS	Eight weeks	Samsung gear sport smartwatch Valid step count compared with the Actigraph (*r* = 0.40)	Physical activity data: daily step counts and daily inactive time Heart rate variability Stress Sleep	SDNN, power, LF/HF ratio increased during the pandemic Decreased step counts, increased daily inactive time and decreased sleep during lockdown
Pépin et al. [28]	France	To determine users' adherence to wearable sensors due to home confinement	Observational	742,000 individuals who used the Withing wearable sensor(Conflicts of interest)	Physical activity data of the registered users were abstracted from the server and analyzed	Not applicable	Wristwatch with the accelerometer (Withings)	Physical activity data (step count) in regional wise distribution	Physical activity in European countries remained two-fold than China Decrease in step count (25—54%) Good compliance with lockdown rules without violating citizens' privacy
Speirs Craig et al. [29]	United Kingdom	to investigate the impact of lockdown on physical activity levels using research grade accelerometers	Secondary analysis from a longitudinal study of 1970 British cohort study	6492 individuals from the British cohort were analyzed	Four valid days of 20 h per day	Not applicable	Thigh mounted triaxial accelerometer (activPAL3) Data for at least 20 h in a day and for four days	Stepping events, Standing Upright events	5797 valid data were analyzed significant increase in median step count (from 2,320 steps to 3,874 steps) for days classified as "indoor only “indoor activity” has found to have lower step count than the “outdoor activity”
Sañudo et al. [30]	Spain	To determine the extent of change in physical activity, sedentary behavior, smartphone use and sleep patterns during the COVID-19 lockdown	Cross-sectional study	22 college students (22.5 ± 2.6 years)	Young adult Aged 20–36 years A resident of the city of Seville	Not applicable	Wristband accelerometer (Xiaomi Mi Band 2, Beijing, China) [ high measurement accuracy concerning HR, steps, distance and sleep; MAP—0.10]	Self-reported physical activity: walking time, MVPA using IPAQ Daily steps count from the wearables	Daily step count reduced during the lockdown Slight increase in total sleep duration during the lockdown Delay in wake time During the lockdown, total physical activity and exercise time reduces There is an urge to leverage the technology-based motion sensor to develop a health promotion protocol at home
Wang et al. [31]	China	To determine any change in daily steps during the pandemic using WeChat To examine the risk factors for poor daily step count during the lockdown	Longitudinal observational study	3544 participants of STEP study who undertake an annual physical check-up at the hospital	Residents of Changsha city aged ≥ 40 years Should have a personal smartphone and have a WeChat account	Two months	Inbuilt accelerometers from smartphones linked to WeChat application	Daily step count was monitored by the phone's inbuilt accelerometer and extracted by WeChat Measured when wear time was > 10 h on a given day low daily step count as ≤ 1500 steps/day	Daily step dropped from 8097 to 5440 steps Prevalence of low step count increased from 3 to 18% Appropriate strategies to actively engage in regular physical activity However, accuracy regarding physical activity is worth mentioning
Woodruff et al. [32]	Canada	To investigate the change in stress, physical activity and screen-related sedentary behavior within the first month of the COVID-19 pandemic (March/April 2020) To identify the barriers associated with the change in physical activity	Survey-based observational study	167 Participants (> 18 years old)	> 18 years of age and older) Regularly using wearables To fill monthly activity calendar	Not applicable	Wearable activity tracker/pedometer (Apple, Fitbit, Samsung, and Garmin)	Objective physical activity variables such as daily step count from activity trackers were self-reported Subjective physical activity time (min/week) Sedentary behavior (screen time and leisure time) Complete survey on physical activity barriers and stress/coping	A significant drop in step count (-2038 steps/day) while self-reported physical activity levels maintained Screen time was also increased substantially Decreased physical activity is found to adversely associated with the work stress

*LF/HF* a ratio between low frequency and high frequency, a measurement variable in heart rate variability, *MET* metabolic equivalent, a measure of energy expenditure, *SDNN* standard deviation of NN interval, a measurement variable in heart rate variability, *USA* United States of America

## Results

**Fig. 1:**
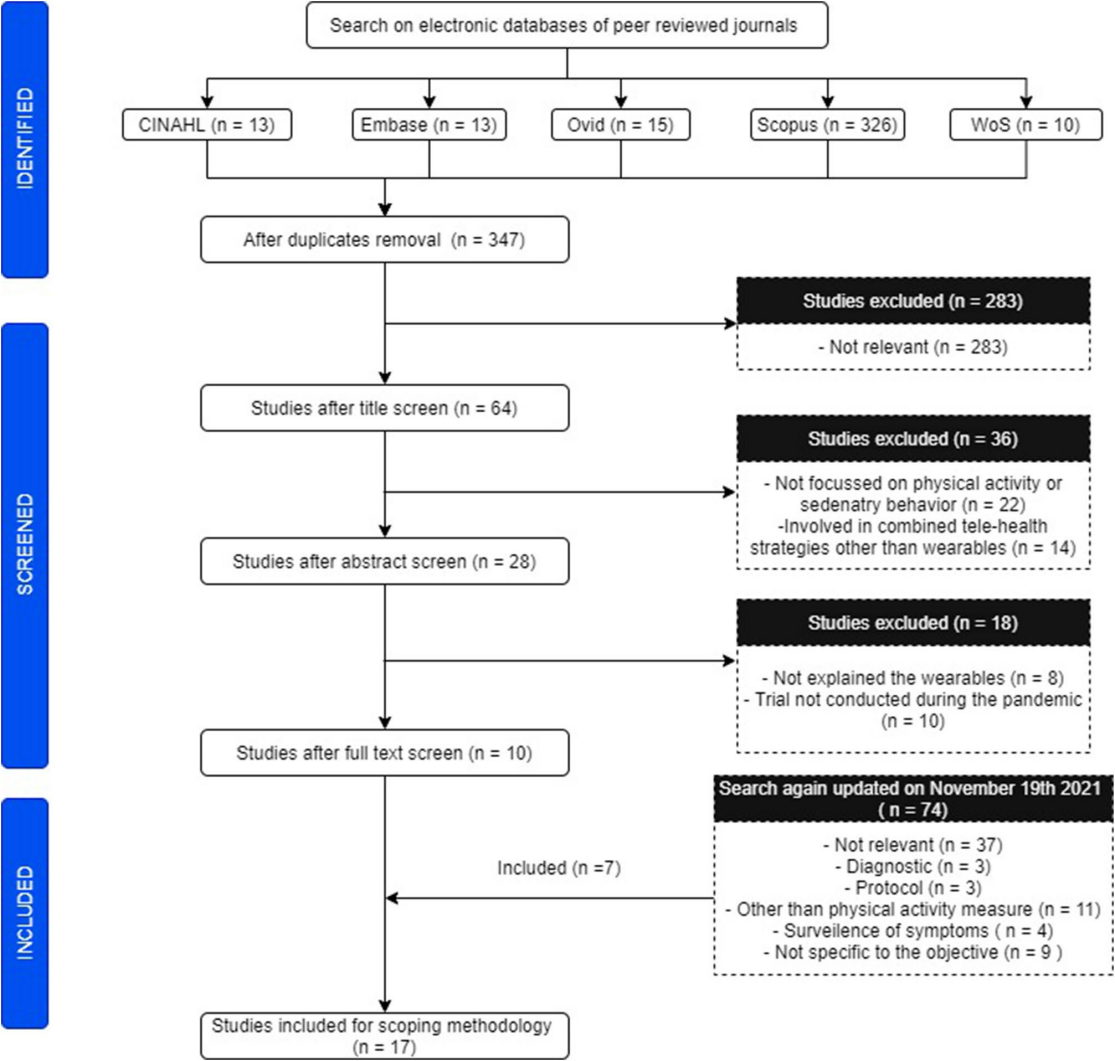
Flowchart of the potential studies screened and included in the review which explored the wearables use for physical activity promotion and sedentary behavior reduction during the pandemic

Of 347 screened, seventeen articles were deemed eligible for inclusion and potential scoping was done. Figure 1 shows the flow of the screening and inclusion of the studies for the review. The majority of the studies (*n* = 14; 82%) included adult participants (*n* = 7,59,979) ranged from 10 to 742,000. Heterogeneity in the study types was observed as follows: cross-sectional (*n* = 9; 53%), longitudinal (*n* = 4; 24%), retrospective (*n* = 1; 6%), narrative review (*n* = 1; 6%) and pilot non-randomized studies (*n* = 2; 12%). Similar heterogeneity was found in participants too in the included studies: pregnant women [27], elderly [24, 26], office workers [21], diabetes [18] and children [25]. All the studies were from high-income countries.

### Wearables and the measurement of physical and physiological variables

Seven studies administered smartphone-based physical activity measurement through inbuilt accelerometers from which the captured data were transferred to the cloud server and visualized in smartphone applications [16–19, 21, 24, 31]. Majority of studies (*n* = 9; 53%) employed wrist bands and wristwatches of multiple technology firms (Apple, Samsung, Xiaomi) and wearable research-based accelerometers [20, 23, 25, 26, 28–30, 32]. A few studies (*n* = 4; 24%) reported the psychometrics of the wearables, and low-to-moderate validity was found [21, 22, 27].

### Physical health measurement through wearables

The mean follow-up period of longitudinal studies was 21 weeks [18, 20, 21, 24, 26, 27]. The average reduction in the daily step count after the pandemic compared to before the pandemic was 2812 steps/day. The studies had reported a significant reduction in standing time (− 32.7%), walking time (− 52.2%) and step count (− 29%) during lockdown when the physical activity was monitored with the wearables [22, 26]. Mean energy expenditure was reduced to 70 kcal compared to before pandemic [23]. Only one study by Vile et al. [27] reported a reduction in heart rate variability variable, especially high LF/HF ratio. Further reduction in step count and sleep time was found to be positively associated with body mass [19], depression [26] and workplace stress [32]. Various behavior change techniques such as self-efficacy, goal setting, prompt/cues, information and social networking were associated with the compliance of wearable use [24, 25, 31].

## Discussion

**Fig. 2:**
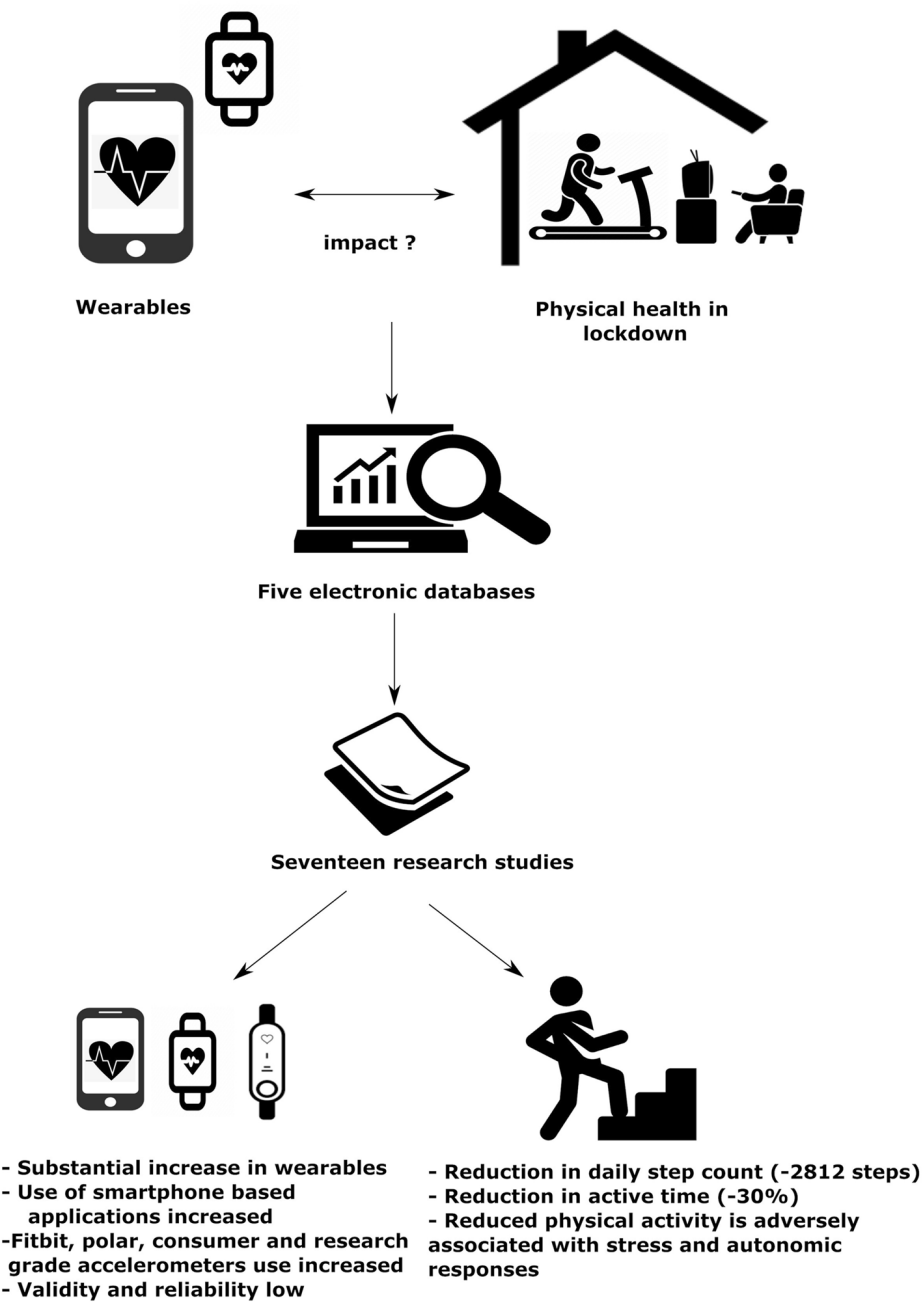
Summary of the scoping review findings

From the evidence included, we found a significant increase in the use of wearables to improve physical activity during the confinement or lockdown periods [21, 24]. There is a convincing evidence to show that there is a significant decrease in physical activity and increased sedentary time in people around the world when judged objectively using wearables [16–32]. Figure 2 represents the summary of our findings. In the following sections, we discuss about: (1) the increased usage of the wearables in spite of the validity and reliability of the phantom algorithms; (2) role of wearables in regulating physical health during the lockdown.

### Use of wearables during COVID-19

Wearables are intriguing means for measuring and monitoring physical activity at the population level in spite of arguments over their validity and reliability [22]. Cloud computing and artificial intelligence have leveraged healthcare through wearable sensors, increasing health monitoring and medical automation for speedy diagnosis, including COVID-19 [33]. The above facts are reciprocated with double-fold increase in utilization of wearable devices to self-monitor physical behavior during this pandemic [16–28, 34]. Although found to be less valid and reliable compared to research-based accelerometers, these wearables provide an opportunity for its end users to self-monitor their physical activity levels, energy expenditure, sleep, sitting time and create a framework for personalized prevention [34]. Our review findings also concur with the above findings from the largest British cohort study, the UK Biobank study [34]. Modern trends in mobile technologies have improved activity recognition and estimation of models making smartphones easily accessible and high accuracy in self-monitoring [35]. Recent wearables and their mobile application interface provide more reliable and easily understandable data visualization, thereby gaining popularity, which is seen in the included studies of our review [28]. Smartwatches, smart bands, smart rings, and bracelets are widespread in human activity monitoring and behavior change [36]. Furthermore, wearables embedded with behavior change techniques such as goal setting, information/counseling, prompts, motivation and social support make wearables a potential choice for increased compliance to behavior interventions and long-term behavior change. We found that the behavioral techniques are least addressed in the studies that have employed the wearables for improving physical activity or reducing sedentary behavior in individuals during the pandemic [21, 24]. We recommend that future wearables be developed along with behavioral scientists to understand target behavior (intensity and type of activity) and incorporate maximum behavior change techniques for higher compliance in community settings [37].

### Physical health regulation with wearables during COVID-19

Our review results showed active time in all age groups is significantly reduced by at least 30–50% after lockdown compared to pre-pandemic periods [16, 17, 19, 21, 22, 25, 26]. Our findings concur with the recent cross-sectional study that investigated smartphone-based physical activity measurement before and after lockdown and found 37% reduction in weekly minutes of PA [38]. Consumer-based wearables allowed users to monitor their activity levels and may potentially improve their compliance toward long-term behavior change, which are increasingly popular during this pandemic. Brickwood et al. (2019) systematically reviewed 28 randomized controlled trials and found a significant increase in step count (standardized mean difference [SMD] 0.24; 95% CI 0.16–0.33), moderate-to-vigorous physical activity (SMD 0.27; 95% CI 0.15–0.39) and energy expenditure (SMD 0.28; 95% CI 0.03–0.54) with wearables use [39]. Our review findings concur with the results of the above review, which concluded that physical activity and active time could be improved significantly with wearable devices. In our review, most of the included studies involved healthy adults and four studies investigated physical activity changes in pregnant women [27], asthmatic children [25] and diabetes population [18, 24]. We could also find a trend of studies exploring the use of wearable technology in the elderly for improving self-efficacy and behavioral change for physical activity promotion [24, 26].

Restriction on sports amenities, public gathering, home confinement and remote work are some of the potential barriers to adequate physical activity practises during this pandemic [40]. We propose wearables to be an intriguing intervention for measuring, advocating and monitoring physical activity for reaping health benefits for being active during this pandemic [41]. Furthermore, the reduction of active time was adversely associated with physical and mental health [42]. The physiological changes such as improved cortisol, a brain-derived neurotrophic factor associated with increased activity time, are postulated to have favorable effects on mental health [43, 44]. Nevertheless, a longitudinal study by Vile et al. [27] found a reduction in the heart rate variability with lower physical activity during lockdown might have unfavorable effects on cardiovascular disease risk. Thus, wearable technology serves as an intriguing means to promote physical activity and reduce sedentary behavior among healthy population and individuals at risk for chronic diseases during these unprecedented times.

### Limitations

Few limitations are worth mentioning: (1) majority of the articles included were conducted between early lockdown (March 2020–June 2020). Hence, the review findings may not represent the intensity of wearables use and their impact on physical activity at present; (2) we observed heterogeneity in terms of age, wearable make and working mechanisms of the Internet of Things and algorithms associated with the cloud transfer and interpretation; (3) furthermore, confounding factors such as financial incentives, personalized coaching, wear time, and multifaceted intervention might be the barriers to establishing the effectiveness of wearables in physical activity advocacy. Hence, our review findings should be interpreted with caution for generalisability; (4) as a scoping review, we aimed to provide an overview or map the existing evidence rather than critically appraising answer to a particular question. Therefore, policy makers should consider the breadth of the existing knowledge rather than depth and provide framework for public health practises when interpreting our review findings. We recommend that future systematic reviews summarize evidence of the impact of wearables on public health that can be translated from research into practice.

## Conclusion

Wearables appear to be impending means of improving physical activity and reducing sedentary behavior remotely during this pandemic. National and organizational policies should adopt wearable technologies to promote physical activity, thereby reducing chronic diseases in the general population.

## Supplementary Information

Below is the link to the electronic supplementary material.

Supplementary file1 (DOCX 25 KB)

## Data Availability

All available data have been presented in the study.
