# Supplementary material for: “Wearables on vogue”: a scoping review on wearables on physical activity and sedentary behavior during COVID-19 pandemic
Source: Sport Sci Health. 2022 Jan 7;18(3):641–57. doi: 10.1007/s11332-021-00885-x (PMC8739535; doi:10.1007/s11332-021-00885-x)
Supplement: Supplementary file 1 — Supplementary file1 (DOCX 25 KB) [file 11332_2021_885_MOESM1_ESM.docx]

**Additional file: Sample search administered on various electronic databases (19^th^ November 2021)**

1. **CINAHL**

| S6 | (("wearable technology" OR wearables OR "wearable devices" OR "wearable sensors" OR wearable OR smartwatch) AND (S1 OR S2)) AND (S3 AND S4 AND S5) |
| --- | --- |
| S5 | S1 OR S2 |
| S4 | "wearable technology" OR wearables OR "wearable devices" OR "wearable sensors" OR wearable OR smartwatch |
| S3 | coronavirus-19 OR covid-19 |
| S2 | sedentary behavior OR sedentary lifestyle OR ( physical inactivity or physically inactive or sedentary ) |
| S1 | physical activity OR ( physical activity or exercise or fitness or physical exercise ) OR ( physical activity or exercise ) |

1. **EMBASE**

No. Query

#4. #1 AND #2 AND #3

#3. 'wearable sensor' OR 'wearable technology' OR

'wearable device'

#2. 'coronavirus disease 2019' OR 'coronavirus

infection' OR 'covid 19'

#1. 'physical activity' OR exercise OR 'physical

exercise' OR 'physical fitness' OR 'sedentary

lifestyle' OR 'sedentary behaviour' OR 'physical

inactivity'

.......................................................

1. **OVID MEDLINE**

| **#** | **Searches** |
| --- | --- |
| 1 | ("physical activity" or "physical fitness" or "physical exercise" or "physical inactivity" or "sedentary behaviour" or "sedentary lifestyle").af. |
| 2 | (covid-19 or Coronavirus-19 or "coronavirus pandemic" or "covid pandemic" or lockdown).af. |
| 3 | ("wearable device" or "wearable sensor" or wearable or smartwatch or wearable technology).af. |
| 4 | 1 and 2 and 3 |

1. **WEB OF SCIENCE**

|  |  |  | | |
| --- | --- | --- | --- | --- |
| # 4 | #3 AND #2 AND #1 | | |  |
| # 3 | **TOPIC:** (coronavirus-19) *OR* **TOPIC:** (covid-19) *OR* **TOPIC:** (lockdown)  *Indexes=SCI-EXPANDED, SSCI, A&HCI, CPCI-S, CPCI-SSH Timespan=1988-2021* | | |  |
| # 2 | **TOPIC:** ("wearable device") *OR* **TOPIC:** ("wearable sensor") *OR* **TOPIC:** (wearable) *OR* **TOPIC:** (smartwatch)  *Indexes=SCI-EXPANDED, SSCI, A&HCI, CPCI-S, CPCI-SSH Timespan=All years* | | |  |
| # 1 | **TOPIC:** ("physical activity") *OR* **TOPIC:** ("physical exercise") *OR* **TOPIC:** ("physical fitness") *OR* **TOPIC:** ("physical inactivity") *OR* **TOPIC:** ("sedentary behaviour") *OR* **TOPIC:** ("sedentary lifestyle")  *Indexes=SCI-EXPANDED, SSCI, A&HCI, CPCI-S, CPCI-SSH Timespan=All years* | |  |  |
